# The fidelity of implementation of recommended care for children with malaria by community health workers in Nigeria

**DOI:** 10.1186/s13012-020-0968-1

**Published:** 2020-03-04

**Authors:** Oluwatomi Adeoti, Donna Spiegelman, Chinenye Afonne, Catherine O. Falade, Ayodele S. Jegede, Frederick O. Oshiname, Melba Gomes, IkeOluwapo O. Ajayi

**Affiliations:** 1 Department of Epidemiology, Harvard T.H. Chan School of Public Health, 677 Huntington Ave, Boston, Massachusetts 02115 USA; 2 Department of Biostatistics, Harvard T.H. Chan School of Public Health, 677 Huntington Ave, Boston, Massachusetts 02115 USA; 3 Department of Nutrition, Harvard T.H. Chan School of Public Health, 677 Huntington Ave, Boston, Massachusetts 02115 USA; 4 Department of Global Health and Population, Harvard T.H. Chan School of Public Health, 677 Huntington Ave, Boston, Massachusetts 02115 USA; 5 Department of Statistics and Data Science, Yale University, New Haven, USA; 6 Center for Methods in Implementation and Prevention Science (CMIPS), Yale School of Public Health, New Haven, USA; 7 Center for Interdisciplinary Research on AIDS, Yale School of Public Health, New Haven, USA; 8 Epidemiology and Biostatistics Research Unit, Institute of Advanced Medical Research and Training (IMARAT), College of Medicine, University of Ibadan, Ibadan, Nigeria; 9 Department of Pharmacology and Therapeutics, College of Medicine, University of Ibadan, Ibadan, Nigeria; 10 Department of Sociology, Faculty of the Social Sciences, University of Ibadan, Ibadan, Nigeria; 11 Department of Health Promotion and Education, Faculty of Public Health, College of Medicine, University of Ibadan, Ibadan, Nigeria; 12 UNICEF/UNDP/World Bank/WHO Special Programme for Research and Training in Tropical Diseases, World Health Organization, Geneva, Switzerland; 13 Department of Epidemiology and Medical Statistics, Faculty of Public Health, College of Medicine, University of Ibadan, Ibadan, Nigeria

**Keywords:** Community health workers, Implementation fidelity, Malaria, Process evaluation, Sub-Saharan Africa

## Abstract

**Background:**

In the context of task shifting, a promoted approach to healthcare delivery in resource-poor settings, trained community health workers (CHWs) have been shown to be effective in delivering quality care of malaria for febrile under-5 children. While their effectiveness has been documented, the fidelity of implementation (FOI) has not been adequately studied. By understanding and measuring whether an intervention has been performed with fidelity, researchers and practitioners gain a better understanding of how and why an intervention works, and the extent to which outcomes can be improved. The objective of this study was to assess the FOI of a recommended protocol for malaria care by CHWs in a resource-poor setting in Nigeria.

**Methods:**

Thirty-five female CHWs who participated in a 3-day training on home management of malaria among under-5 children were studied. They managed 1,646 children over the implementation period and then underwent evaluation via a one-time hospital-based observation by the trainers. During the evaluation, a pre-tested standard checklist was used to compute performance scores for CHWs; doctors and nurses were selected to serve as the gold standard for comparison. Performance scores (PS) recorded during the evaluation were used to assess adherence and compliance with the recommended treatment protocol.

**Results:**

Of the 4 skill domains assessed, adherence was greatest for compliance with malaria treatment recommendations (94%) and lowest for post-treatment initiation counseling of home-based caregivers (69%). The average overall adherence of 83% was comparable to adherence by gold standard comparators. Mean PS was not found to be significantly associated with CHW demographics. Scores for clinical evaluation among those whose occupation was not healthcare-related were significantly lowered by 0.52 [95% CI (1.05–0.01), *p* = 0.05]. Compliance with the treatment protocol increased by 23% for every unit increase in total PS (*p* = 0.07) and doubled for every unit increase in scores for post-treatment initiation counseling of caregivers (*p* = 0.002).

**Conclusions:**

Studying intervention fidelity stands to identify the shortcomings of implementation and specific areas to target for improvement in future adoption or implementation. This study concludes that future trainings should emphasize clinical evaluation and post-treatment counseling of caregivers by CHWs to ensure the best outcome for children.

Contributions to the literature

- Consolidates existing evidence that CHWs should be included in primary healthcare provision, especially in resource-poor settings.
- Highlights that training of CHWs should emphasize better interaction with patients and their caregivers, especially as related to eliciting signs and symptoms of diseases that they are trained to manage and continued care of the patient beyond the clinic setting.
- Emphasizes the need for implementers to agree on measures of fidelity before beginning implementation to enable an exhaustive examination of multiple factors that can influence it.
- Provides reference for future researchers who will carry out similar implementation research in Nigeria, where the field is upcoming.

## Background

Children younger than the age of 5 years are highly susceptible to recurrent febrile illness. In sub-Saharan Africa (SSA), malaria makes a significant contribution to the burden of febrile illness among these children. Reportedly, it is the fourth leading cause of under-5 mortality in sub-Saharan Africa [1]. In 2016, there were an estimated 216 million cases of malaria and 445,000 malaria deaths in 91 countries. Africa had the highest burden of malaria: 90% of malaria cases and 91% of malaria deaths (2016) [2]. In addition to malaria being one of the leading causes of under-5 mortality in the world, the United Nations 2017 report on levels and trends in child mortality highlights the fact that children in sub-Saharan Africa are about 15 times more likely to die before the age of 5 than children in high-income countries [3].

The first of the three pillars of the WHO Global Technical Strategy for Malaria 2016–2030 is to ensure universal access to malaria prevention, diagnosis, and treatment [4]. Many community-based interventions have been successfully implemented by community health workers over the years, with their effectiveness in reducing child morbidity and mortality well demonstrated [5]. Home management of malaria (HMM) is a simple and effective intervention that puts malaria drugs into the hands of mothers and community-based caregivers. Community-based caregivers may include community health workers (CHWs) and community medicine distributors (CMDs); CHWs are members of a community who undergo short-term training to provide a specific preventive, curative, or rehabilitative care to their community. The availability of antimalarial treatment near the home and in the community has proven to significantly reduce malaria morbidity and mortality in children and to increase equity in access [6, 7]. In addition, the current best practice recommendation is that all cases of suspected malaria should have a parasitological test (microscopy or a rapid diagnostic test—RDT) to confirm the diagnosis [8]. Adopting this practice in home management of malaria reduces the over-diagnosis of malaria cases and unnecessary use of antimalarial drugs, both of which have contributed to the development of antimalarial drug resistance [2]. In this study, the context-adapted protocol implemented by the CHWs included early and accurate diagnosis of malaria using clinical signs and symptoms, confirming diagnosis with rapid diagnostic tests (RDTs), prompt treatment of uncomplicated malaria with artemisinin-based combination therapy (ACT) and pre-referral treatment of severe malaria with artemisinin-based suppositories (rectal artesunate—RA) [8, 9].

Fidelity of implementation (FOI) is a component of implementation research, a type of research that focuses on evaluating the process involved in executing health interventions with proven effectiveness. FOI is defined in the literature as the degree to which providers implement programs as intended by the program developers [10]. Only by evaluating whether an intervention has been adopted with fidelity can researchers and practitioners gain a better understanding of how and why an intervention works, and the extent to which outcomes can be improved [11].

A conceptual framework for FOI described by Carroll et al. (2007) identifies 5 measurable elements of FOI from research published between 2002 and 2007: adherence to intervention, exposure/dose, quality of delivery, participant responsiveness, and program differentiation. Two subjective elements were also included in the framework: intervention complexity and facilitation strategies [11]. This framework considers adherence to be the bottom-line measurement of implementation fidelity, increased by exposure/dose—the extent to which the elements of an intervention were delivered to its recipients as prescribed by its designers. Quality of delivery is defined as the way the intervention providers deliver a program, using techniques prescribed by the program or those suggested by similar programs. Participant responsiveness measures the degree to which participants respond to, or are engaged by, an intervention. Program differentiation refers to identifying unique features of different components or programs and identifying which elements of programs are essential, without which the program will not have its intended effect, i.e., what aspects of the program are redundant and what aspects are essential for its success. Intervention complexity identifies facilitators and barriers to the adoption of an intervention. Facilitation strategies are put in place to optimize the level of fidelity achieved. Such strategies included the provision of manuals, guidelines, training, monitoring and feedback, capacity building, and incentives. All these elements are included as potential moderators that link intervention to adherence.

The importance of fidelity in program implementation cannot be overemphasized. If fidelity is linked with the outcome, the intervention can be modified to amplify outcomes, improve efficiency, and can be explored for generalizability. In health studies, literature establishes that higher fidelity scores are associated with better health outcomes, ranging from patient satisfaction to the efficient delivery of services by health care workers [12, 13]. CHWs are key to the effective delivery of primary health services, particularly in resource-poor and rural areas [14]. Therefore, it is of value to evaluate the fidelity of primary healthcare interventions delivered by CHWs if their usefulness in healthcare delivery is to advance.

Our study is a process evaluation embedded within a multisite malaria intervention in 3 sub-Saharan African countries namely: Burkina Faso, Nigeria, and Uganda. One of the main goals of the larger effectiveness evaluation by Ajayi et al. (2016) was to assess the feasibility of malaria diagnosis and treatment by CHWs in rural areas of malaria-endemic countries in Africa. The project team performed pre-post intervention surveys to determine the impact of the intervention on the study population. The results of the effectiveness study showed that in all 3 intervention sites, the intervention resulted in a significant increase in the number of caregivers who visited trained CHWs to obtain care for their sick wards as well as a significant increase in RDT diagnosis of malaria and administration of ACTs [15]. Of the 3 countries, we focused on the Nigerian site because of the accessibility of the data at the time this process evaluation was completed. In Nigeria, CHW visits, RDT diagnosis, and ACT administration improved by 10.4%, 51.4%, and 32.1% respectively.

The objective of this study was to assess the fidelity of implementation of the recommended protocol for malaria care by CHWs in a resource-poor setting within SSA as well as the influence of sociodemographic characteristics on CHW performance during the implementation phase of the intervention. The evidence generated will provide a basis for the replication of this targeted health intervention in similar settings, that is, the inclusion of trained CHWs in the diagnosis and treatment of malaria, especially in areas where access to health facilities is limited.

## Methods

### Aim and setting of the study

The intervention evaluated in this study was designed and implemented by a university research team sponsored by the UNICEF-UNDP-World Bank-WHO Special Program for Research and Training in Tropical Diseases (TDR) between December 2013 and October 2015. The project was titled—Feasibility, acceptability, and costs of a community-based diagnostic and treatment package for malaria of varying degrees of severity in sub-Saharan Africa. It was a 3-phase single-arm non-randomized multi-center community-based intervention carried out in selected rural malaria-endemic areas of Burkina Faso, Nigeria, and Uganda [15]. The primary objective was to assess the feasibility, acceptability, and cost-effectiveness of a package for the diagnosis and treatment of uncomplicated and severe malaria in children under 5 years of age delivered at the community level.

### Design of the study

The intervention was designed to be entirely implemented by CHWs. Therefore, the project team set out to select, train, and supervise the delivery of the recommended care by the CHWs.

#### Selection of CHWs

Intensive advocacy and sensitization of community heads, opinion leaders, and residents of the selected communities were carried out in the company of the local government primary healthcare unit coordinator and malaria control program officers. The community heads were then requested to select those to be trained as CHWs from their communities. Selection criteria for CHWs included being a permanent resident (lived at least 1 year in the current location), trusted and respected by the community, able to keep simple records, spousal consent as applicable, and a willingness to serve. The number of trainees selected per community was based on the population of the community. One trainee was selected per population of approximately 50 people. Everyone selected was interviewed by the investigators to ascertain their suitability or otherwise, those who did not meet the selection criteria were replaced with the assistance of the community heads.

#### Training of CHWs

**Fig. 1:**
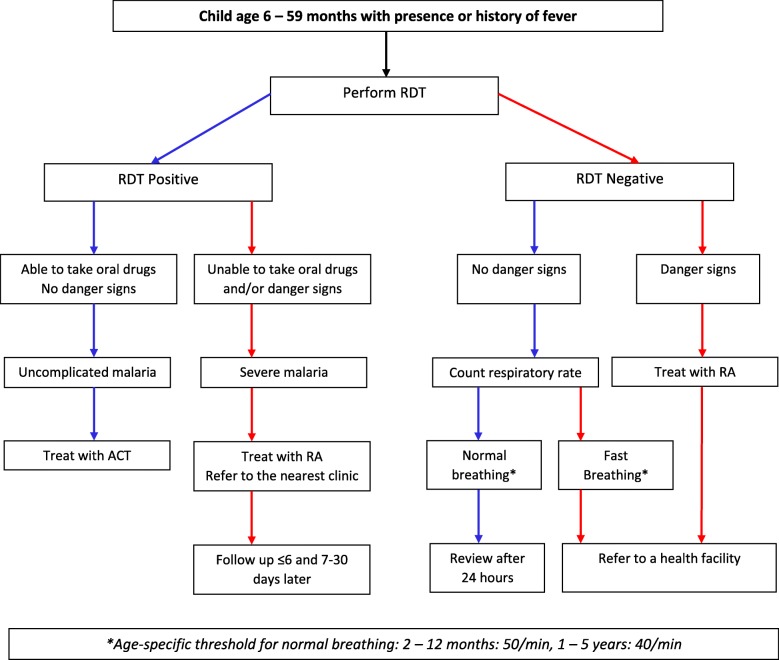
Intervention diagnostic and treatment protocol. Source: Ajayi I. O, Nsungwa-Sabiiti J. N, Traore A. Gomes M, Pagnoni F. Feasibility, acceptability and costs of a community-based diagnostic and treatment package for malaria of varying degrees of severity in sub-Saharan Africa. Clinical study protocol submitted to UNICEF-UNDP-World Bank-WHO Special Program for Research and Training in Tropical Diseases (TDR), 2010. Protocol code: MAL-TDR_08 Project ID A80550

In Nigeria, 55 female volunteers were invited to participate in a 3-day training by the project team on this community-based intervention. The intervention protocol was designed for CHWs to recognize symptoms and signs of complicated and uncomplicated malaria early, confirm diagnosis with malaria RDTs, treat and refer when indicated, identify signs of other common causes of febrile illness in under-5 children, and counsel caregivers after treatment initiation. Figure 1 shows the diagnostic and treatment protocol taught to and implemented by the trained CHWs. Pre- and post-training interactive sessions and role-play were key components of the training program. All trainers contributed by sharing their observations and providing feedback to participants. In so doing, comprehensiveness of the training was assessed and corrections were made where necessary. Quarterly refresher training was also done. See details of the training here—Training Community Health Workers to Manage Uncomplicated and Severe Malaria: Experience From 3 Rural Malaria-Endemic Areas in Sub-Saharan Africa [16].

#### Supervision of CHWs

Supervision was done in-person and remotely. Telephone calls to all the CHWs were made every morning on weekdays. On weekends, CHWs were able to contact the supervisory team via a dedicated phone line to make urgent requests. Visitation was prioritized in the following order: those with blood smear samples, those who could not be reached due to cell phone network problems, then all others, resulting in at least two visits per week to each implementation site. Activities carried out during visitations included replenishment of medical consumables, collection of prepared blood smear slides, verification of positive RDT results, inspection of records kept, and correction of observed errors.

#### Evaluation of CHWs’ performance*.*

Fifty female volunteers completed all the training sessions. Ten of these were judged incompetent due to their inability to perform the RDTs and keep records as taught during the training. Thus, a satisfactory competency level was achieved by 80% of participants; all 40 were recruited as CHWs for the implementation phase of the intervention. During the implementation phase, 5 of these performed below expectation especially relating to record-keeping and availability at their assigned implementation site for care administration, supervisory visits, and/or calls by the project team. This study evaluated the 35 CHWs who maintained minimum competency standards in the field.

After 22 months of implementing the intervention, the 35 CHWs underwent a performance evaluation. Evaluation was via a one-time hospital-based observation of each CHW managing a child with febrile illness by the investigators/trainers. During the evaluation, a pre-tested standard checklist was used to compute performance scores for the CHWs and medical personnel selected to serve as the gold standard for comparison. These medical personnel were doctors and nurses who had ample experience treating malaria in under-5 children and had been part of the training team for the CHWs. Hence, they were well acquainted with the intervention protocol. The total performance score was computed as a sum of scores obtained in all four domains assessed—general practice, proficiency in the use of RDT, adherence to malaria treatment recommendation, and post-treatment initiation counseling of caregivers.

### Source of data

This study involved the analysis of data from the 35 CHWs who managed 1,646 children and were evaluated in the Ona-Ara local government area of Oyo state located in Southwest Nigeria. Performance scores recorded during the evaluation were used as a measure of adherence to the implementation protocol developed for the intervention. Records of malaria RDT results and treatment administered to the 1,646 children managed by the 35 CHWs were also utilized.

### Explanatory variables, outcome variables, and covariates

The analysis was done in two parts. The first part focused on the influence of the sociodemographic characteristics of CHWs (explanatory variables) on adherence to the recommended management protocol as determined by the performance score (outcome variable). In the second part, we examined the relationship between CHW performance score during evaluation (explanatory variable) and compliance with the intervention treatment protocol (outcome variable coded as 1 if the correct treatment was administered to the child based on the RDT result and 0 otherwise). Sociodemographic characteristics of CHWs (age, level of education, and occupation) were included as confounders in this second part of the analysis. The occupation was reported as having or not having a healthcare-related job, such as a community birth attendant or a patent medicine seller.

### Data analysis

Means (SD) were presented for continuous variables and frequencies (%) for categorical variables. The frequency distribution of CHWs’ sociodemographic characteristics, average performance score (total and by domain), percentage adherence to each step in the protocol, and overall adherence were reported. Percentage adherence for the comparison group was also reported. Percentage adherence per domain was defined as the sum of the percentages of CHWs (or comparators) who executed each step in a domain divided by the total number of steps in each domain. Having established normality of the outcome variable—performance scores (Shapiro-Wilk test *p* value > 0.05), *t* test, and ANOVA were used to compare scores across CHWs’ sociodemographic groups. For the first part of the analysis, linear regression was used to model the association between performance scores and sociodemographic characteristics. For the second part of the analysis, the association between compliance with treatment and performance scores of CHWs was modeled using logistic regression, adjusting for CHWs’ age, level of education, and occupation. Differences in performance scores, odds ratios, and attendant 95% confidence intervals were presented. All analyses were performed with SAS version 9.4 (SAS Institute, Cary, NC), using an α level of 0.05 to assess significance.

## Results

The mean age of the participants was 42.0 (9.4) years. Table 1 shows the frequency distribution and mean performance scores across sociodemographic groups of the CHWs. Both total and domain performance scores are shown. Table 2 displays percentage adherence to the intervention protocol by the CHWs relative to the comparators. The average overall adherence was 82.8% and comparable to adherence by gold standard comparators (82.5%). Of the 4 skill domains assessed during the evaluation, adherence was lowest for the domain that assessed post-treatment initiation counseling of caregivers (69.3%); highest for the use of the malaria rapid diagnostic test kit (91.1%) and for compliance with malaria treatment recommendation (93.6%). The adherence rates per domain were higher among CHWs versus nurse and physician comparators except in one domain—general practice. Table 3 shows the cross-sectional CHW sociodemographic correlates of performance scores. Though mean total performance scores were higher among younger CHWs, among those with more than the primary level of education, and among those who had a healthcare-related job, none of these sociodemographic characteristics were found to be significantly associated with performance. For the domain that assessed clinical evaluation of patients (general practice), those whose occupations were not healthcare-related had significantly lower performance scores in both crude [− 0.56 (− 1.06 to − 0.06), *p* = 0.03] and adjusted [− 0.52 (− 1.05–0.01), *p* = 0.05] models. There were no other differences in mean performance scores by sociodemographic characteristics at the α = 0.05 level.

**Table 1:** Distribution of performance scores by CHW characteristics (*n* = 35)

Variable (range)	*n* (%)	Domain performance score: mean (SD)				Total score (18–26)^a^
		General practice (1–3)^a^	Proficiency in the use of RDT (8–13)^a^	Adherence to malaria treatment recommendation (5–6)^a^	Post-treatment initiation counseling of caregivers (1–4)^a^	
Age quartiles in years						
1st (23–33)	6 (17.1)	2.5 (0.6)	12.0 (5.7)	5.7 (0.5)	2.7 (1.5)	22.8 (2.9)
2nd (35–40)	12 (34.3)	2.7 (0.7)	10.9 (1.4)	5.7 (0.5)	3.0 (0.9)	22.3 (2.1)
3rd (42–47)	8 (22.9)	2.3 (0.7)	11.1 (1.7)	6.0 (0.0)	3.0 (0.8)	22.4 (2.0)
4th (48–65)	9 (25.7)	2.1 (0.8)	10.8 (1.2)	5.7 (0.5)	2.3 (1.3)	20.9 (1.8)
Age categories in years						
≤ 40 years	18 (51.4)	2.6 (0.6)	11.3 (1.5)	5.7 (0.5)	2.9 (1.1)	22.4 (2.3)
> 40 years	17 (48.6)	2.2 (0.7)	10.9 (1.4)	5.8 (0.4)	2.7 (1.1)	21.6 (2.0)
Highest level of education						
Primary	13 (37.1)	2.3 (0.8)	10.5 (1.5)	5.9 (0.4)	2.9 (1.0)	21.5 (1.9)
Secondary	18 (51.4)	2.4 (0.7)	11.6 (1.3)	5.7 (0.5)	2.6 (1.2)	22.3 (2.4)
Post-secondary	4 (11.4)	2.5 (0.6)	11.0 (1.4)	5.8 (0.5)	3.0 (0.8)	22.3 (1.5)
Occupation						
Healthcare-related	10 (28.6)	2.8 (0.4)	11.0 (1.5)	5.7 (0.5)	2.8 (0.9)	22.3 (2.5)
Not healthcare-related	25 (71.4)	2.2 (0.7)	11.2 (1.5)	5.8 (0.4)	2.8 (1.2)	21.9 (2.0)
Mean performance score	–	2.4 (0.7)	11.1 (1.5)	5.7 (0.4)	2.8 (1.1)	22.0 (2.2)

Mean performance score = Total score points obtained by CHW per domain/35

^a^Range of performance score

**Table 2:** Adherence to program protocol by CHWs compared to gold standard

Domain	% Adherence^c^	
	CHW	Gold standard (comparator)
A. General practice	77.2	79.0
i. Asked about fever	88.6	87.1
ii. Asked for child’s age	100	100
iii. Temperature measurement taken^a^	5.9	12.5
iv. Respiratory rate measurement^b^	4.0	0.0
v. Looked for danger signs (signs of severe malaria)	42.9	50.0
B. Proficiency in the use of rapid diagnostic test kit	91.1	90.6
i. Checked for safety measure	85.7	86.2
ii. Labeled the cassettes used for each child	97.1	85.7
iii. Pricked the child’s finger correctly	88.6	90.3
iv. Accurate blood volume collected	77.1	83.9
v. Correct volume of buffer applied	97.1	96.6
vi. Blood and buffer placed in wells appropriately	91.4	89.7
vii. Waited specified time before reading the results	94.3	96.3
viii. Interpreted the results correctly	97.1	96.4
C. Adherence to malaria treatment recommendation	93.6	92.5
i. Told caregiver the result of the RDT		
ii. RDT positive—gave artemether-lumefantrine (ACT)	74.3	70.0
iii. or rectal artesunate (RA) as required	100	100
iv. RDT negative—did not give ACT or RA	100	100
v. Gave the correct dose of ACT or RA when applicable	100	100
D. Post-treatment initiation counseling of caregivers	69.3	67.9
i. Correct medication dosage	97.1	97.1
ii. Told caregiver how to use oral medications	68.6	68.6
iii. Ask to return within 48 h if the child’s health does not improve	54.3	50.0
iv. Told the caregiver to feed the child well	57.1	55.9
Average Adherence	82.8	82.5

^a^51% missing, not included in mean score and % adherence for Domain A

^b^29% missing, not included in mean score and % adherence for Domain A

^c^% Adherence per domain = (sum of % of CHWs or comparators who executed each step in a domain/total number of steps in each domain)

**Table 3:** Association between demographics of CHWs and performance scores

	Crude		Adjusted^a^	
	Change in mean score (95% CI)	*P* value	Change in mean score (95% CI)	*p* value
Dependent variable: total performance score				
Age in years	−0.04 (−0.12–0.04)	0.30	−0.04 (− 0.12–0.05)	0.36
Highest level of education	0.20 (− 0.32–0.73)	0.43	0.18 (− 0.36–0.72)	0.50
Occupation				
Healthcare-related	Ref		Ref	
Not healthcare-related	−0.38 (−2.03–1.27)	0.64	−0.08 (−1.84–1.67)	0.93
Dependent variable: performance score for general practice				
Age in years	−0.02 (− 0.04–0.01)	0.15	−0.01 (− 0.03–0.01)	0.31
Highest level of education	0.00 (− 0.17–0.17)	1.00	−0.04 (− 0.20–0.13)	0.64
Occupation				
Healthcare-related	Ref		Ref	
Not healthcare-related	−0.56 (− 1.06 – − 0.06)	0.03*	−0.52 (− 1.05–0.01)	0.05*
Dependent variable: performance score for proficiency in the use of RDT				
Age in years	− 0.03 (− 0.09–0.02)	0.20	−0.04 (− 0.09–0.02)	0.17
Highest level of education	0.25 (− 0.10–0.60)	0.15	0.26 (− 0.09–0.62)	0.14
Occupation				
Healthcare-related	Ref		Ref	
Not healthcare-related	0.16 (−0.98–1.30)	0.78	0.56 (− 0.64–1.66)	0.38
Dependent variable: performance score for adherence to malaria treatment recommendation				
Age in years	0.005 (− 0.01–0.02)	0.55	0.004 (− 0.01–0.02)	0.61
Highest level of education	−0.01 (− 0.12–0.10)	0.82	−0.008 (− 0.12–0.11)	0.89
Occupation				
Healthcare-related	Ref		Ref	
Not healthcare-related	0.06 (−0.28–0.40)	0.72	0.03 (− 0.34–0.40)	0.86
Dependent variable: performance score for post-treatment initiation counseling of caregivers				
Age in years	0.007 (− 0.03–0.05)	0.72	0.008 (− 0.04–0.05)	0.71
Highest level of education	−0.03 (− 0.30–0.23)	0.79	−0.04 (− 0.32–0.24)	0.79
Occupation				
Healthcare-related	Ref		Ref	
Not healthcare-related	−0.04 (− 0.88–0.80)	0.92	−0.10 (− 1.01–0.80)	0.82

^a^Adjusted for age (years), highest level of education (primary, secondary, post-secondary), and occupation of CHW (healthcare-related, not healthcare-related)

*Significant at *p* < 0.05

Of the 1,646 children treated by these 35 CHWs, 1,621 received the correct treatment based on their RDT results, giving treatment compliance of 99% during implementation. Table 4 presents the association between compliance with treatment and performance scores of CHWs modeled using logistic regression. The model adjusted for the age, level of education, and occupation of CHW yielded an OR of 1.25 (95% CI 0.95–1.65, *p* = 0.11). For the domain that assessed post-treatment initiation counseling of caregivers, the OR for the association between compliance with treatment and performance score was 2.16 (95% CI 1.32–3.53, *p* = 0.002) in the unadjusted model and 2.00 (95% CI 1.24–3.22, *p* = 0.004) after adjusting for age, level of education, and occupation of CHWs.

**Table 4:** Association between performance scores of CHWs and compliance with treatment

Independent variable (performance score)	Crude		Adjusted^a^	
	OR (95% CI)	*p* value	OR (95% CI)	*p* value
Total score	1.23 (0.98–1.53)	0.07	1.25 (0.95–1.65)	0.11
General practice	1.46 (0.68–3.14)	0.33	0.95 (0.38–2.37)	0.92
Proficiency in the use of RDT	1.04 (0.72–1.50)	0.84	0.98 (0.59–1.62)	0.93
Adherence to malaria treatment recommendation	0.74 (0.17–3.31)	0.70	0.75 (0.17–3.45)	0.72
Post-treatment initiation counseling of caregivers	2.16 (1.32–3.53)	0.002*	2.00 (1.24–3.22)	0.004*

Per the intervention protocol, compliance with treatment was defined as the correct treatment of a RDT positive child without danger signs with artemisinin-based combination therapy (ACT), a RDT positive child with danger signs with rectal artesunate (RA) followed by referral for further management, and not treating an RDT negative child with either ACT or RA

^a^Adjusted for age (years), highest level of education (primary, secondary, post-secondary), and occupation of CHW (healthcare-related, not healthcare-related)

*Significant at *p* < 0.05

## Discussion

We assessed the fidelity of implementation of a context-adapted diagnostic and treatment protocol for children under 5 years of age, using post-intervention performance evaluation scores and compliance with recommended treatment during fieldwork as measures. Age, level of education, and primary occupation of the community health workers did not significantly affect almost all aspects of performance. This supports the assertion by WHO that community health workers can be men or women, young or old, literate or illiterate [14]. In this study, adherence to the diagnostic and treatment protocol by trained CHWs was found to be better than that of the comparative medical personnel on average, suggesting that with adequate training, CHWs can effectively carry out their assigned duties. Adherence was highest for the use of the malaria RDT kit and for compliance with the malaria treatment recommendation. These are in keeping with other studies conducted in similar settings where CHWs used RDT testing to diagnose malaria and administered treatment to under-5 children [17, 18]. Recent emphasis on the need for large-scale community health worker programs highlights the recognition of their effect on strengthening health systems and fostering equity in health access [19, 20]. They provide a channel worth exploring to address the predicted global shortage in health workers by 2030 [21].

The lack of an association between sociodemographic characteristics and adherence to protocol in this group of CHWs could be because the training, refresher training, and supervision of CHWs were effective during the intervention. Almost all CHWs had visits from field supervisors at least twice in a week during which all aspects of their work were reviewed and corrections made as needed. Existing literature confirms that training, re-training, and close supervision of CHWs ensure the effectiveness of the interventions that they deliver to their communities [22–24]. Our study also showed that there was a 23% increase in the odds of compliance with treatment for a one-unit increase in performance score, though this was not statistically significant possibly due to lack of power (*p* = 0.07). Despite these encouraging results, future studies that explore the sustainability of these bolstering mechanisms after the intervention has been established will help evaluate the long-term effectiveness of the intervention. If CHWs need to receive repeated trainings to ensure effectiveness, modalities of this must be factored into interventions that rely mostly on CHWs [25].

In terms of the content of trainings, our study revealed a dearth in two aspects of performance. First, those without prior experience with the administration of any form of medical care had significantly lower scores for the domain that assessed clinical evaluation of patients (general practice). Though this suggests that there may be an advantage in recruiting existing patent medicine sellers and community birth attendants as CHWs, doing this will reduce the number of people that are available for this kind of community-based intervention. Rather, more emphasis should be placed on better training of CHWs without prior healthcare experience. WHO has established that literacy is not a hindrance to working as a CHW; more so, being a member of the community they serve promotes commitment [14]. The second domain of performance that showed the need for more focus during the trainings was post-treatment initiation counseling of caregivers. In this domain, there was a significant doubling in the odds of compliance for every unit increase in performance scores. These results, in addition to the fact that percentage adherence was lowest for this domain, necessitates an improvement in this aspect of care offered to the children. The role of caregivers in ensuring that patients comply with treatment initiated by trained healthcare workers after they leave the controlled treatment setting cannot be overemphasized. Training mothers to administer home management of malaria has been shown to significantly improve their children’s outcomes [6, 26]. Adherence to dosage recommendations, when to return to the clinic if no improvement in symptoms is observed, and referral instructions are vital aspects of post-treatment initiation counseling that should be emphasized to home-based caregivers. Neglecting these activities increases the risk of child morbidity, increases the risk of developing drug resistance with poor dosing compliance, and can lead to loss of confidence in the CHW if the child does not improve.

Despite the significant results observed, we identified some limitations to our study. In addition to demographic characteristics, other factors that might have influenced the fidelity of implementation were not measured and so, could not be adjusted for during analysis. Carroll et al. (2007) recommended examining the influence of facilitation strategies in greater detail, strategies such as the number of trainings that the CHWs participated in. In this study, the evaluation used to assess fidelity was done towards the end of the implementation period. Performance scores obtained via objective assessment of CHWs at multiple time points over the implementation period would have contributed valuable information to the findings in this study and mitigated the potential Hawthorne effect on the assessment of CHW performance during the one-time hospital-based evaluation. Other elements of FOI not accounted for in this study include quality of delivery, participant responsiveness, program differentiation, and intervention complexity. Though sometimes difficult to measure, some of these can have a significant impact on the fidelity of implementation. During the design phase, implementers should consider and agree on indicators of implementation fidelity so these can be measured during the implementation phase of the intervention. In addition to this, greater power to detect more associations between protocol compliance and performance scores could have occurred if the number of wrongly treated children was greater than 17 out of the 1,646 children that received treatment. That is, a larger all-sample size under the same 99% compliance will provide better statistical power, especially for the adjusted association. Lastly, this study is limited to descriptions of FOI among CHWs who were able to successfully complete the training and demonstrate minimum competency standards after completion. Further research can entail exploring ways of increasing the success rate of the training and field competency of CHWs.

## Conclusion

Adherence to a community-based diagnostic and treatment protocol for malaria by CHWs is comparable to that of experienced formal healthcare providers like doctors and nurses. Irrespective of age, level of education, and type of primary occupation, trained CHWs performed well in providing the recommended care for children. This is congruent with the results of the effectiveness evaluation of the intervention. Future trainings should emphasize methods for clinical assessment of patients, especially for those who have never administered medical care as well as post-treatment counseling of caregivers, to ensure the best outcomes for children. The scale-up potential of this intervention is supported by findings in this study and should be leveraged to increase access to recommended care for malaria in endemic parts of the world, especially in resource-poor settings where access to comprehensive primary care is limited.

## Data Availability

The data that support the findings of this study are available from UNICEF-UNDP-World Bank-WHO Special Program for Research and Training in Tropical Diseases (TDR), but restrictions apply to the availability of these data, which were used under license for the current study, and so are not publicly available. Data are, however, available from the authors upon reasonable request and with permission of UNICEF-UNDP-World Bank-WHO Special Program for Research and Training in Tropical Diseases (TDR).

## Abbreviations

ACT: Artemisinin-based combination therapy
CHW: Community health worker
CMD: Community medicine distributor
FOI: Fidelity of implementation
HMM: Home management of malaria
IRC: Institutional Review Committee
RA: Rectal artesunate
RDTs: Rapid diagnostic tests
SSA: Sub-Saharan Africa
WHO: World Health Organization
